# Glioma management and outcome in low-middle-income countries: a systematic review

**DOI:** 10.3389/fonc.2026.1856016

**Published:** 2026-06-12

**Authors:** Adam M. Abdallah, Atef F. Hulliel, Asem A. Almomani, Rawhi Alshaykh, Omar H. Abuhashem, Sara Khaled Aldalki, Tala Y. Dabash, Mohammad Mukahal, Ala’ Marji, Said Mahmoud Lahham, Mouness Obeidat

**Affiliations:** 1 Neuro Oncology Surgery, Department of Surgery, King Hussein Cancer Center, Amman, Jordan; 2 Department of Neurosurgery, Brain and Spine, Morsani College of Medicine, University of South Florida, Tampa, FL, United States; 3 Faculty of Medicine, Jordan University of Science and Technology, Irbid, Jordan; 4 Department of Radiation Oncology, King Hussein Cancer Center, Amman, Jordan

**Keywords:** glioma, LMIC, management, outcome, systematic review

## Abstract

**Background:**

Gliomas are the most common primary malignant brain tumors, yet evidence on their management and outcomes in low- and middle-income countries (LMICs) remains limited. Disparities in diagnostic capacity, treatment access, and healthcare infrastructure create unique challenges that are poorly characterized in the existing literature.

**Method:**

A systematic review was conducted following PRISMA guidelines and registered in PROSPERO (CRD420261359813). PubMed and Scopus were searched for studies reporting glioma management and outcomes in LMICs. Two independent reviewers performed screening, data extraction, and quality assessment using the Newcastle-Ottawa Scale.

**Results:**

Thirty studies comprising 3,463 patients from 17 low- and middle-income countries (LMICs) were included. Low-grade gliomas accounted for 62.6% of tumors, while high-grade gliomas represented 37.4%. Molecular profiling was reported in 26.7% of studies. LGGs showed significantly better outcomes than HGGs, with pooled 5-year OS rates of 87.8% and 21.9%, respectively.

**Conclusion:**

Glioma management in LMICs is characterized by limited access to adjuvant therapies, minimal molecular diagnostic integration, and heterogeneous survival outcomes that fall below standards achieved in high-income countries. Targeted investments in radiotherapy infrastructure, chemotherapy access, and molecular diagnostic capabilities are essential to reduce global disparities in neuro-oncological care.

**Systematic Review Registration:**

https://www.crd.york.ac.uk/PROSPERO, identifier CRD420261359813.

## 1 Introduction

Gliomas are the most common primary intracranial tumors, representing 81% of malignant brain tumors (1). They arise from the neoplastic transformation of glial cells, including astrocytes, oligodendrocytes, and ependymal cells, and encompass a heterogeneous group of neoplasms with widely varying biological behavior and clinical outcomes (2). Glioblastoma represents the most common primary malignant brain tumor with an incidence rate of 3.23 per 100,000 population (3). The prognosis of glioma patients remains poor; less than 3% of glioblastoma patients are still alive at 5 years after diagnosis, with higher age being the most significant predictor of poor outcome (4). Overall survival differs markedly among glioma subtypes, with 5-year survival rates ranging from approximately 94.7% in pilocytic astrocytoma to just 6.8% in glioblastoma (3).

Management of gliomas relies on a multimodal strategy incorporating surgery, radiotherapy, and chemotherapy. Surgical resection remains the cornerstone of initial treatment, with the extent of resection serving as a key prognostic factor. Radiotherapy is also a fundamental component of cancer care, in high-income settings, approximately 52% of newly diagnosed patients require at least one course of radiotherapy, and up to 25% may need a second course (5).

In high-income countries, glioma care follows a well-defined, structured pathway, from imaging to surgery, radiotherapy, and targeted therapy. In contrast, in most low- and middle-income countries (LMICs), this pathway is often fragmented and delayed, shaped by factors that extend well beyond the tumor itself.

Despite the growing body of research on glioma biology and treatment, there is still a notable lack of summarized evidence addressing the distinct challenges of glioma care in LMICs. Understanding disparities in diagnosis, management, and outcomes in these settings is essential to guide targeted improvements in neuro-oncological care globally. Therefore, this systematic review aims to comprehensively evaluate glioma management in LMICs by providing a broad overview of current practices rather than generating subtype-specific treatment recommendations, with a focus on patient demographics, treatment strategies, access to adjuvant therapies and molecular diagnostics, and survival outcomes.

## 2 Method

This systematic review was performed according to Preferred Reporting Items for Systematic Reviews and Meta-analyses (PRISMA) guidelines (6). The protocol was registered in the PROSPERO database (CRD420261359813).

Ethical approval and informed consent of patients were not needed because we only collected data from previously published studies and did not recruit patients.

### 2.1 Eligibility criteria and study selection

We included studies meeting the following criteria:

The population of interest comprised all patients diagnosed with glioma (including low-grade glioma, benign glioma, diffuse astrocytoma, oligodendroglioma, pilocytic astrocytoma, subependymal giant cell astrocytoma, pleomorphic xanthoastrocytoma, angiocentric glioma, chordoid glioma, gangliocytoma, and ganglioglioma) in low- and middle-income countries (LMICs), with inclusion of all age groups, encompassing both pediatric and adult populations. The intervention and comparator categories included any management strategies reported in these settings, including surgical interventions (gross total resection, subtotal resection, biopsy), radiotherapy, chemotherapy, and conservative or supportive management approaches.

For study outcomes, we required that at least one of the following results was reported: survival outcomes (overall survival, event-free survival, progression-free survival), functional or clinical outcomes, follow up, treatment-related complications, extent of resection, or recurrence rates. Additionally, outcomes related to challenges in glioma management in resource-limited settings, including delays in diagnosis, limited access to adjuvant therapies, and availability of molecular diagnostics, were considered.

We considered retrospective or prospective observational study designs, including cohort studies, case series, cross-sectional studies and registry-based analyses. Randomized controlled trials, if available, were also eligible for inclusion. Case reports, editorials, conference abstracts without full-text availability, letters to the editor, and review articles were excluded.

### 2.2 Search strategy and keywords

A comprehensive and systematic search was conducted across two major electronic databases, PubMed and Scopus, to identify relevant studies on glioma management in low- and middle-income countries.

The PubMed search strategy utilized a combination of Medical Subject Headings (MeSH) terms and free-text keywords to maximize sensitivity. The search string was as follows: [(“Developing Countries”[Mesh] OR low-income OR middle-income OR global OR “limited resource*” OR LMIC*)] AND [(low-grade glioma* OR benign glioma* OR diffuse astrocytoma* OR oligodendroglioma* OR pilocytic astrocytoma* OR subependymal giant cell astrocytoma* OR pleomorphic xanthoastrocytoma* OR angiocentric glioma* OR chordoid glioma* OR gangliocytoma* OR ganglioglioma OR Glioblastoma)].

The Scopus search employed an analogous strategy using equivalent free-text terms: (developing countries OR low-income countries OR middle-income countries) AND (low grade glioma OR benign glioma OR diffuse astrocytoma OR oligodendroglioma OR pilocytic astrocytoma OR Glioblastoma).

The search was supplemented by manually scanning reference lists of all retrieved full-text articles to identify additional eligible studies not captured by the electronic database searches.

Studies that did not match the above criteria were excluded from the analysis. Two independent reviewers blindly screened the exported search results in Rayyan (Qatar Computing Research Institute, QCRI). Rayyan is a free online semi-automated screening tool developed by QCRI to assist in archiving, organizing, and selecting articles for systematic reviews. Studies were screened for eligibility through two sequential steps: (1) title and abstract screening for studies matching the inclusion criteria; and (2) full-text articles of eligible abstracts were retrieved and screened for final eligibility. Conflicts were resolved through discussion and consensus between the two reviewers, and when consensus could not be reached, a third reviewer was consulted.

### 2.3 Data extraction

Data extraction was performed independently by three authors using standardized extraction tables and online collaborative spreadsheets (Google Sheets) to ensure consistency and facilitate cross-verification. Discrepancies were resolved through discussion among the reviewers.

The following data were extracted from each included study: first author name, country of origin, study design, year of publication, diagnosis (glioma subtype), total number of patients, gender distribution (male/female), and age in years (reported as mean, median, or range as available).

Treatment-related data were extracted, including the type and count of surgical interventions (gross total resection, subtotal resection, biopsy), number of patients receiving radiotherapy, and number of patients receiving chemotherapy. Outcome data extracted included follow-up duration (in years), event-free survival (EFS), and overall survival (OS).

### 2.4 Quality assessment

Two independent reviewers blindly assessed the methodological quality of the included studies using the Newcastle-Ottawa Scale (NOS), adapted for case series/cohort studies. The NOS evaluates studies across three domains: Selection (maximum 4 stars), Comparability (maximum 2 stars), and Outcome (maximum 3 stars), yielding a total score out of 9 stars. Studies scoring 7–9 stars were classified as high quality (low risk of bias), those scoring 4–6 stars as moderate quality (some concerns), and those scoring 0–3 stars as low quality (high risk of bias). Overall, the included studies demonstrated a moderate methodological quality. The total NOS scores ranged from 3 to 8 stars, with a median score of 6.0. A detailed breakdown of the quality ratings revealed that the majority of studies (n=18, 60%) were of moderate quality. Ten studies (33.3%) were rated as high quality, while only two studies (6.7%) were classified as low quality. Although the NOS was originally designed for cohort and case-control studies, it has been widely adapted for quality assessment of case series in systematic reviews of surgical literature. We acknowledge that alternative tools, such as the Joanna Briggs Institute (JBI) Critical Appraisal Checklist for Case Series, may offer a more tailored framework for non-comparative studies. However, the NOS was selected to maintain consistency with previously published reviews and to enable comparability across included studies with varying designs. The inherent limitation of applying NOS to single-arm studies is reflected in the low Comparability domain scores, as most included studies lacked external comparison groups.

### 2.5 Dealing with missing data

The statistical formulas of Wan et al. were used to calculate the mean and standard deviation (mean ± SD) when some studies reported results using the median with range (7).

### 2.6 Ethical approval

Ethical approval and informed consent of patients were not required because we only collected data from previously published studies and did not recruit patients.

### 2.7 Statistical analysis

Pooled estimates of 5-year overall survival were calculated using a random-effects meta-analysis model with Freeman–Tukey double arcsine transformation to stabilize variances of proportions near the boundaries of 0 and 1. Study-specific weights were assigned using inverse variance weighting. Between-study heterogeneity was quantified using the I² statistic and Cochran’s Q test, with I² values of 25%, 50%, and 75% considered indicative of low, moderate, and high heterogeneity, respectively. Subgroup analyses were performed by tumor grade (low-grade vs. high-grade glioma) and population type (pediatric vs. adult). Weighted least-squares meta-regression was conducted to explore potential moderators of heterogeneity, including publication year and proportion of high-grade glioma patients. Publication bias was assessed using funnel plot visual inspection and Egger’s regression test for funnel plot asymmetry. Robustness of the pooled estimates was evaluated through leave-one-out sensitivity analysis. For demographic variables (age, sex distribution), pooled weighted means were calculated using study sample sizes as weights, with means and standard deviations estimated from medians and ranges using the method of Wan et al. when necessary. Treatment proportions (surgery, radiotherapy, chemotherapy) were calculated as aggregate rates by summing event counts and denominators across reporting studies. All statistical analyses were performed using R software and statistical significance was set at p < 0.05.

## 3 Result

### 3.1 Literature search results

**Figure 1:**
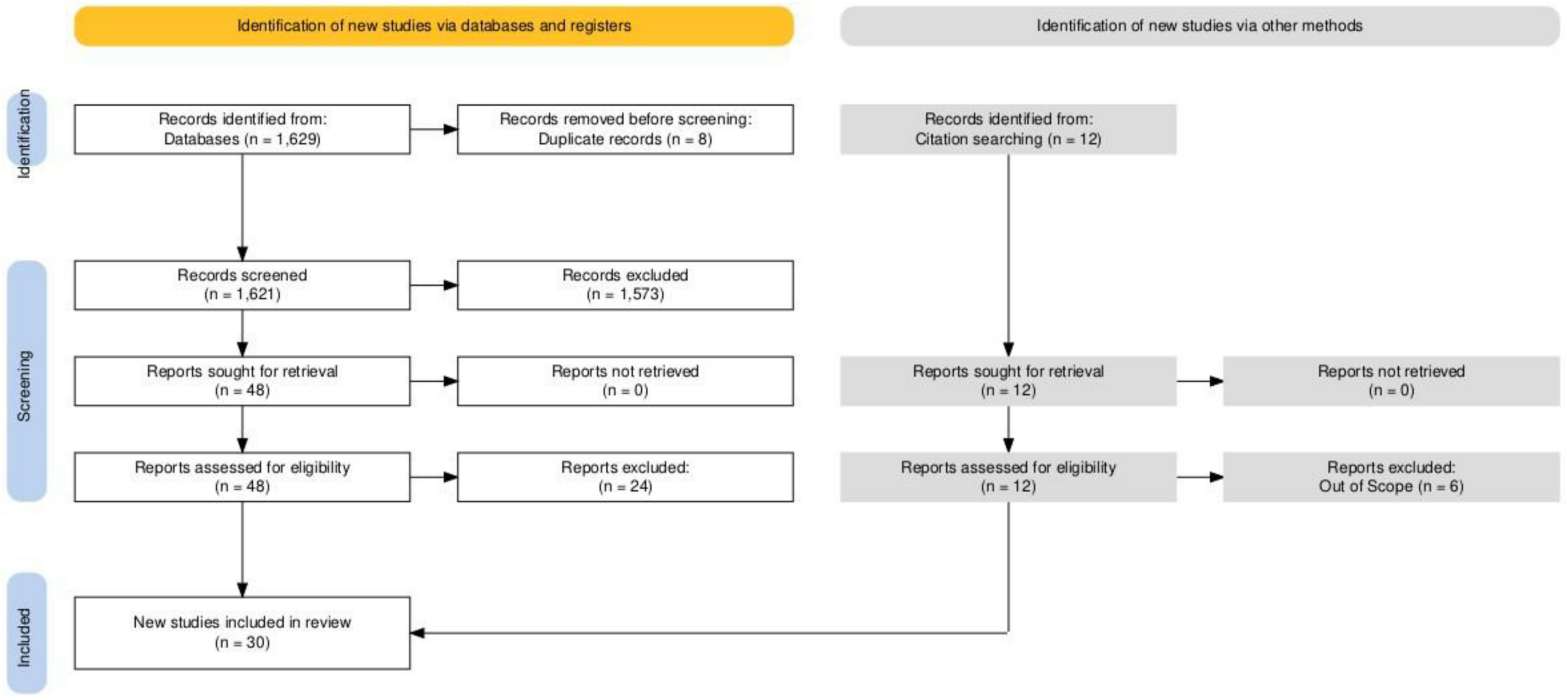
PRISMA flow chart.

Our search yielded 1608 PubMed results, while the Scopus search identified 21 documents, producing a combined total of 1629 records. After removal of 8 duplicates, 1621 unique records remained for screening. Following title and abstract screening, 48 articles were selected for full-text retrieval. Of these, 43 full-text articles were successfully retrieved and assessed for eligibility. After applying the inclusion and exclusion criteria to the full-text articles, 24 (8–37) studies met all criteria and were included in the final systematic review (see the PRISMA flow diagram in **Figure 1**). Additionally, we performed manual reference extraction from the selected studies and added 6 more articles, which are included in the final count of 30 studies.

### 3.2 Study characteristics

A total of 30 studies were included in the final analysis, encompassing data from 17 countries and representing a cumulative cohort of 3,463 patients. The included studies demonstrated substantial methodological heterogeneity, with a predominance of observational designs.

Most studies employed retrospective methodologies, including retrospective cohort studies, institutional reviews, and registry-based analyses. Prospective designs were less frequently reported. Additional study types included cross-sectional analyses, descriptive case series, and diagnostic-focused investigations, each contributing a smaller proportion to the overall dataset.

Sample sizes varied considerably, ranging from small single-center cohorts to larger multicenter or registry-based populations. This variation reflects differences in institutional capacity, case volume, and data availability across settings.

Geographically, the dataset was composed of studies from low- and middle-income countries, consistent with the study objective. However, there was broad regional diversity, contributing to variability in healthcare infrastructure, diagnostic capabilities, and treatment availability. A summary of the design and main findings of the included studies is given in **Table 1**.

**Table 1:** A summary of the design and main findings of included studies.

Study ID	Study design	Study period	Country	No. of participants	Gender (M/F)	Age	Surgical intervention type and count	Radiotherapy no.	Chemotherapy no.	Follow-up (years)	Event-free survival	Overall survival
Bajwa et al.	Retrospective cohort	2023	Pakistan	281	196/85	N/A	Biopsy: 47; STR: 163; GTR: 71	143 (RT) + 100 (CRT)	RT+PCV: 85; RT+TMZ: 15; Chemo: 40	N/A	IDH-mut astro mean prog: 27.9 mo; 1p19q co-del oligo: 55.5 mo	IDH-mut astro: 32.2 mo; 1p19q co-del oligo: 54.2 mo; 1p19q co-del GTR: 91.7 mo
Beltrán et al.	Retrospective cohort	2018	Mexico	99	53/46	Mean 44 ± 16	GTR: 18; STR: 35; Biopsy: 46	34 (RT) + 13 (CRT) = 47	13 (CRT with TMZ)	2.5 (30 mo)	N/A	GI: 100% at 30 mo; GII: 88%; GIII: 0%; GIV: 0%
Khan et al.	Retrospective cohort	2016	Pakistan	16	11/5	Median 37 (23-62)	Awake craniotomy (n=16)	2 (RT only)	12 (CRT)	NR	N/A	N/A
Benyaich et al.	Retrospective cohort	2015	Morocco	20	14/6	Mean 34 ( ± 10.5)	Awake craniotomy with mapping (n=20)	N/A	N/A	0.5 (6 mo)	N/A	N/A
Abu Laban et al.	Retrospective cohort	2024	Jordan	20	13/7	Median 8.3 (0.3-18.9)	Partial resection: 10; Biopsy: 9; No surgery: 1	N/A	Chemo before targeted Rx: 14; BRAF/MEK inhibitors: 20	LGG 1.9; HGG 2.7	LGG: all partial/stable response; HGG: all progressed	LGG: all alive; HGG: 2 died
Ramírez-Melo et al.	Retrospective cohort	2024	Mexico	30	11/19	Median 6 (0.3-14.1)	Biopsy: 11; Resection: 9	6 (20%)	12 (40%); carbo ± vincristine	Median 5	5y EFS: 79.3% ± 10.8%; 10y: 61.7%	5y OS: 89.5% ± 6.9%; 10y: 71.6%
Stanić et al.	Retrospective cohort	2021	Serbia	173	90/83	Mean 8.96 (1-18)	GTR: 62; STR: 47; Reduction: 34; Biopsy: 10	164 (94.8%)	116 (67.1%)	Mean 7.9 (94.5 mo)	N/A	LGG 5y OS: 90.5%; HGG 5y OS: 9.5%
Sahara et al.	Retrospective cohort	2021	Indonesia	75	44/31	Mean 44.8 (2-73)	Surgical removal (n=75)	N/A	N/A	N/A	N/A	N/A
Ordoñez-Rubiano et al.	Retrospective cohort	2025	Colombia	272	122/150	Mean 48.8 ± 21.0	NTR: 109 (40.1%); STR: 101 (37.0%); GTR: 31 (11.5%); Biopsy: 31 (11.5%)	N/A	N/A	Mean 0.98 (11.8 mo)	N/A	GBM 2y: 21%; Glioma NOS 5y: 38%; Astro 5y: 15%; Oligo 8y: 5%
Mushtaq et al.	Retrospective cohort	2025	Pakistan	47	19/28	Median 11 (IQR 8-16)	GTR: 10; STR: 37	7 (RT); 4 (RT+CCNU+TMZ); 6 (RT+CCNU); 9 (RT+combos)	9 (CCNU); 8 (TMZ); combos	Median 0.59	N/A	1y OS: 29.8%; 2y OS: 23.4%; CMMRD+ 2y: 20%; CMMRD− 2y: 25%
Mosaab et al.	Retrospective cohort	2020	Egypt	105	59/46	Mean 7.54	Biopsy (tumor samples)	N/A	N/A	Average 2	Median EFS (mut): GII 5.4 mo, GIII 8.5 mo, GIV 3.6 mo	Median OS (mut): GII 7.9 mo, GIII 11.6 mo, GIV 7.2 mo; DIPG 6.5 mo
Amayiri et al.	Retrospective cohort	2023	Jordan	32	16/16	Median 9.5 (0.9-21.5)	Biopsy/resection (all); 21 at dx, 11 at progression	~5	~13 (chemo before targeted Rx)	Median 0.87 (10.4 mo)	N/A	LGG: 15/15 alive; HGG: 6/10 alive, 4 dead
Khalid et al.	Retrospective cohort	2025	Pakistan	101	53/48	Mean 16.9 ± 12.7; Peds: 59, AYA: 35, Adult: 7	GTR: 37 (43%); NTR: 7 (8.1%); STR: 38 (44.1%); Biopsy: 4 (4.6%)	4 (RT); 8 (CRT)	4 (chemo); 8 (CRT)	Median OS: Peds 5.02, AYA 5.79, Adult 4.41	1y PFS: 50%; 5y PFS: 10%; Median PFS: Peds 1.55, AYA 2.08, Adult 2.43	1y OS: 92%; 5y OS: 87%; 10y OS: 83%
Stagno et al.	Retrospective cohort	2014	Uganda	172	103/69	Mean 6.5 (1 mo-19 y)	Craniotomy (n=172); Biopsy: 20	N/A	N/A	Mean 1.89 (22.7 mo)	N/A	5y OS: 59.8%
Mahmoud et al.	Retrospective cohort	2021	Egypt	23	12/11	Mean 6.6 (1-17)	NTE: 20 (87%); STE: 3 (13%)	4 (17.4%; with chemo)	6 (26.1%; 4 with RT, 2 alone)	Mean 0.98 (11.8 mo)	No progression observed	1 death (4.3%)
Shah et al.	Retrospective cohort	2024	Pakistan	285	201/84	Mean 43.44 ± 15.1	STR: 188 (66%); GTR: 29 (10.2%); Biopsy: 68 (23.9%)	265 (93.0%)	141 (PCV or TMZ)	N/A	N/A	Mean OS: GBM 14.1 mo, AA 27.5 mo, AO 39.8 mo; by surgery: biopsy 11.8, STR 19.6, GTR 40.2 mo
Mwachaka et al.	Retrospective cohort	2025	Kenya	84	52/32	Median 8 (1-18)	GTR: 49 (58.3%); STR: 27 (32.1%); Biopsy: 8 (9.5%)	3 (3.6%)	2 (2.4%)	Median 4.36 (survivors)	N/A	6 mo: 74%; 1y: 67%; 3y: 56%; 5y: 46%; Median OS: 3.86 y
Baquero-Herrera et al.	Retrospective cohort	2022	Colombia	7	5/2	Median 5 (1-12)	Partial resection: 5 (71.4%); Biopsy: 2 (28.6%); ETV: 3	7 (100%; 3 also CRT)	3 (with RT)	Median 0.06 (23 d)	N/A	Median OS: 23 days (7-52 d)
Hoveyan et al.	Retrospective cohort	2024	Armenia	149	84/65	Mean 6.24, median 7 (3 mo-25 y)	GTR: 15 (15.5%); STR: 17 (17.5%); Partial: 2; Biopsy: 5; Unknown: 58	31 (20.8%)	33 (22.2%)	5y OS reported	N/A	LGG 5y OS: 80%; Median OS not reached. HGG median OS: 13 mo
de Araujo et al.	Retrospective cohort	2011	Brazil	103	55/45	Mean 7.6 ( ± 3.6)	Complete resection: 41; Incomplete/biopsy: 62	63 (61.2%)	90 (chemo)	N/A	N/A	5y: 45% (all); LG astro: 84%; Ependymoma: 33%; HG astro: 0% at 50 mo
Becker et al.	Retrospective cohort	2010	Brazil	31	16/15	Mean 7.8 ± 4.2 (1-17)	GTR: 23 (74.1%); Incomplete: 8	N/A	2 (postop chemo)	Mean 5.7 ± 5.4	5y PFS: 55% (GTR: 70%, partial: 42%)	5y OS: 93.5%; 2 deaths (6.4%)
Fawzy et al.	Retrospective cohort	2018	Egypt	227	124/103	Median 6 (1-18)	Total/NTR: 105; STR: 49; Biopsy: 55; Radiological dx: 18	8 (3.5%; salvage)	99 (43.6%)	1-5	3y EFS: 65.5%; Surg only: 77.3%; Adj chemo: 65.1%	3y OS: 87.3%; Surg only: 95.2%; Adj chemo: 87.4%
Khan et al.	Retrospective cohort	2012	Pakistan	22	11/11	Mean 9.25	Max resection: 15 (68.2%); Partial: 5 (22.7%); Biopsy: 2 (9.1%)	N/A	NR	Mean 3.72 (0.25-12)	N/A	Inpatient mortality: 4.5%; Late mortality: 1 death at 8.4 y
Mehrvar et al.	Retrospective cohort	2014	Iran	198	124/74	Mean 6.11 ± 3.65 (1-14)	Biopsy/partial/total (varying by tumor type)	Varying by type (e.g., MB 77.2%, ependymoma 80%)	Varying by type (e.g., MB: CCNU+Vcr+Dexa; LGG: Carbo+Vcr)	Mean 1.75 (21 mo)	N/A	Survival rate: 51.7% ± 5.2%; 82 deaths (41.4%); 11 LTFU (5.6%)
Nikitovic et al.	Retrospective cohort	2011	Serbia	212	133/79	Mean 9.7 ( ± 4.5, 2.5-18)	Surgical resection (all patients)	212 (100%)	91 (42.9%)	Mean 3.9 (46.9 mo)	N/A	LG astro: 5y OS 92.4%, 8y OS 92.4%; Malignant gliomas: 5y OS 47.8%
Papusha et al.	Retrospective cohort	2020	Russia	69	N/A	N/A (0-18 y)	Complete resection: V600E 4, WT 8; STR/biopsy: V600E 11, WT 46	N/A	N/A	Median: V600E 1.9, WT 1.6	2y PFS: V600E 30.0%; WT 66.2%	N/A
Sevilla-Castillo et al.	Retrospective cohort	2018	Mexico	21	11/10	Mean 7 (2-12)	Partial resection: 12; Total: 8	7 (33.3%)	17 (81.0%)	5	N/A	5y OS: 76%
Sharma et al.	Retrospective cohort	2004	India	23	16/7	Mean 13.2 (4-37)	Total resection: 9; STR: 14	7	N/A	Mean 3.09 (37.1 mo)	N/A	2 postop deaths (8.7%); remaining alive at FU
Varan et al.	Retrospective cohort	2007	Turkey	98	52/46	Median 9 (1-16)	Total: 14; STR: 68; Biopsy: 16	82 (84%)	47; no chemo: 51	Median 5.2 (62.2 mo)	5y EFS: 55%; Overall EFS: 45.7%	5y OS: 62%; LGG: 93.3%; HGG: 22.4%
Pongtanakul et al.	National registry study	2020	Thailand	468	P1: 180/120; P2: 91/77	P1: 7.27 ( ± 4.0); P2: 6.86 ( ± 4.34)	N/A	N/A	N/A	N/A	N/A	P1 10y OS: 39.2% (LGG 69.2%, HGG 14.0%); P2 5y OS: 60.4% (LGG 75.7%, HGG 32.7%)

LGG, low-grade glioma; HGG, high-grade glioma; GBM, glioblastoma; DIPG, diffuse intrinsic pontine glioma; SEGA, subependymal giant cell astrocytoma; PA, pilocytic astrocytoma; OPG, optic pathway glioma; AA, anaplastic astrocytoma; AO, anaplastic oligodendroglioma; GTR, gross total resection; STR, subtotal resection; NTR, near-total resection; NTE, near-total excision; STE, subtotal excision; RT, radiotherapy; CRT, chemoradiotherapy; TMZ, temozolomide; PCV, procarbazine/CCNU/vincristine; CCNU, lomustine; Carbo, carboplatin; Vcr, vincristine; Dexa, dexamethasone; ETV, endoscopic third ventriculostomy; NR, not reported; N/A, not available/applicable; mo, months; y, years; d, days; FU, follow-up; LTFU, lost to follow-up; dx, diagnosis, CMMRD; constitutional mismatch repair deficiency; IDH, isocitrate dehydrogenase; mut, mutant; WT, wild-type; prog, progression; MB, medulloblastoma.

### 3.3 Patient demographics

**Figure 2:**
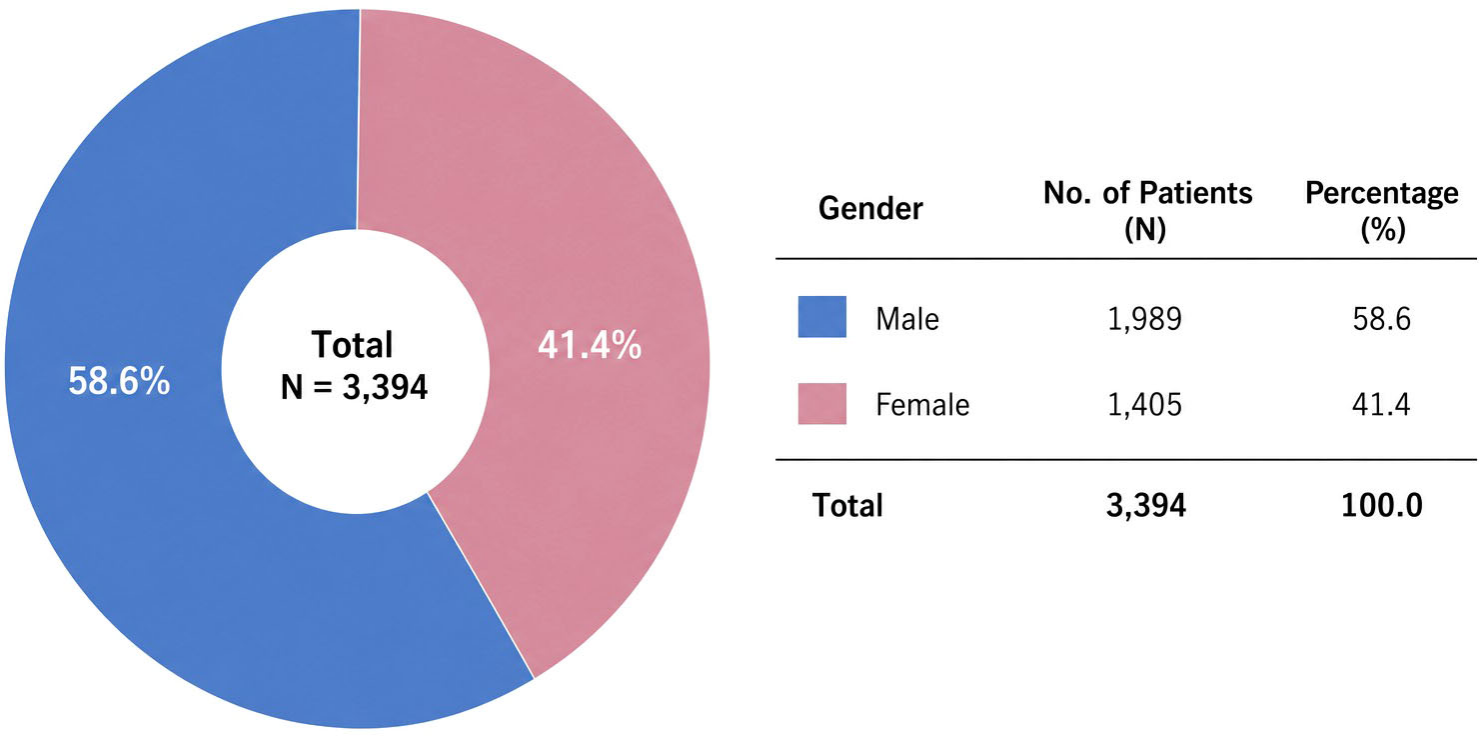
Sex distribution of patients.

Across the included studies, a total of 3,463 patients were analyzed. Sex data were available for 3,394 patients across 29 studies; one study (2) did not report sex distribution. Overall, males comprised 1,989 patients (58.6%) and females 1,405 (41.4%) (**Figure 2**), yielding a male-to-female ratio of 1.42:1. Male predominance was observed in 24 of 29 reporting studies (82.8%). The degree of male predominance varied by population: it was most pronounced in adult studies (M: F 1.68:1; 657 males, 391 females across 6 studies, 1,048 patients) and more moderate in pediatric studies (M:F 1.32:1; 1,263 males, 959 females across 20 studies, 2,222 patients). Two mixed-age studies showed the lowest ratio (M: F 1.25:1; 69 males, 55 females, 124 patients).

Age reporting was heterogeneous, with studies providing means, medians, and ranges in various formats. After standardization using the methods of Wan et al. and weighting by study sample size, the pooled mean age across 28 studies with extractable age data (3,113 patients) was 17.1 years. This relatively young pooled age reflects the predominance of pediatric-focused studies in the review (21/30, 70.0%). The overall reported age range extended from 1 (20) to 73 years (14), indicating inclusion of both neonatal and elderly populations.

When stratified by population type, the weighted mean age was 7.5 years across 21 pediatric studies (2,346 patients) and 45.1 years across 7 adult studies (973 patients), with 2 studies including mixed-age populations (Khalid et al., mean 16.9 years; Sharma et al., mean 13.2 years). Within pediatric cohorts, most studies reported median ages between 5 and 9 years. Several studies documented a substantial proportion of very young children: Stagno et al. reported 29% of patients younger than 2 years (20), Fawzy et al. reported 34.7% younger than 5 years (29), and Mwachaka et al. reported 19% aged 0–4 years (23). Among adult studies, mean ages ranged from 34 years (11) to 48.8 years (15). Within HGG-specific adult cohorts, age varied by histological subtype: Shah et al. reported mean ages of 46.98 years for GBM, 37.17 years for anaplastic astrocytoma, and 44.03 years for anaplastic oligodendroglioma (22).

Predisposing genetic conditions were reported in a subset of studies. Neurofibromatosis type 1 (NF-1) was documented in 23.3% of patients in the Ramírez-Melo et al. optic pathway glioma cohort and in one patient in the Becker et al. series. Sharma (35) et al. focused exclusively on subependymal giant cell astrocytoma in the setting of tuberous sclerosis complex. Mushtaq et al. identified constitutional mismatch repair deficiency (CMMRD) in 31.9% of pediatric HGG patients, with 93% of CMMRD-positive families reporting consanguinity (16). The high prevalence of consanguinity-associated genetic tumor predisposition syndromes in certain LMIC populations (particularly South Asia and the Middle East) represents a distinct epidemiological feature not commonly encountered in high-income country datasets.

### 3.4 Tumor characteristics

Among 28 studies with tumor grade data, 1,352 patients (62.6%) had low-grade gliomas (LGG; WHO grade I-II) and 808 (37.4%) had high-grade gliomas (HGG; WHO grade III-IV). Nine studies focused exclusively on LGG (868 patients), two on HGG (332 patients), and ten reported mixed-grade cohorts. Among LGG studies, pilocytic astrocytoma was the most common subtype, followed by optic pathway glioma, diffuse astrocytoma, and subependymal giant cell astrocytoma. Among HGG studies, glioblastoma was the most frequent, followed by anaplastic astrocytoma and diffuse midline glioma.

Molecular characterization was reported in 8 of 30 studies (26.7%). The most commonly tested markers included BRAF (V600E mutations and KIAA1549: BRAF fusions), IDH1/2, H3K27M, MGMT promoter methylation, and constitutional mismatch repair deficiency. Several findings were clinically significant: Mosaab et al. demonstrated that H3K27M overrode histological grading (HR 3.44 for OS); Amayiri et al. found targetable alterations in 59% of pediatric CNS tumors by next-generation sequencing, and BRAF V600E-positive LGG showed lower 2-year PFS (30% vs 66% wild-type, p = 0.001) but responded to targeted therapy.

### 3.5 Treatment modalities

Surgical intervention was the most consistently reported modality. Among 29 studies with surgical data, 2,842 of 2,995 patients (94.9%) underwent surgery. In approximately 22 studies with extent-of-resection data: GTR was achieved in 27.7%, STR/NTR in 52.9%, and biopsy alone in 19.4%. The low GTR rate reflects late presentation, limited intraoperative navigation, eloquent tumor locations, and restricted surgical expertise in many LMIC settings.

Radiotherapy was administered to 1,025 of 1,913 patients (53.6%) across 18 studies. Chemotherapy was administered to 855 of 2,048 patients (41.7%) across 20 studies, with temozolomide the most referenced agent for HGG. Access to adjuvant therapies varied substantially, with RT rates from 3.5% to 100% and chemotherapy rates from 2.9% to 81.0% across individual studies (**Table 2**).

**Table 2:** Treatment Modalities.

Treatment	n/N	Rate
Surgery	2,842/2,995	94.9%
Gross total resection	574/2,069	27.7%
Subtotal resection/NTR	1,094/2,069	52.9%
Biopsy only	401/2,069	19.4%
Radiotherapy	1,025/1,913	53.6%
Chemotherapy	855/2,048	41.7%

### 3.6 Survival outcomes

#### 3.6.1 Overall 5-year survival

**Figure 3:**
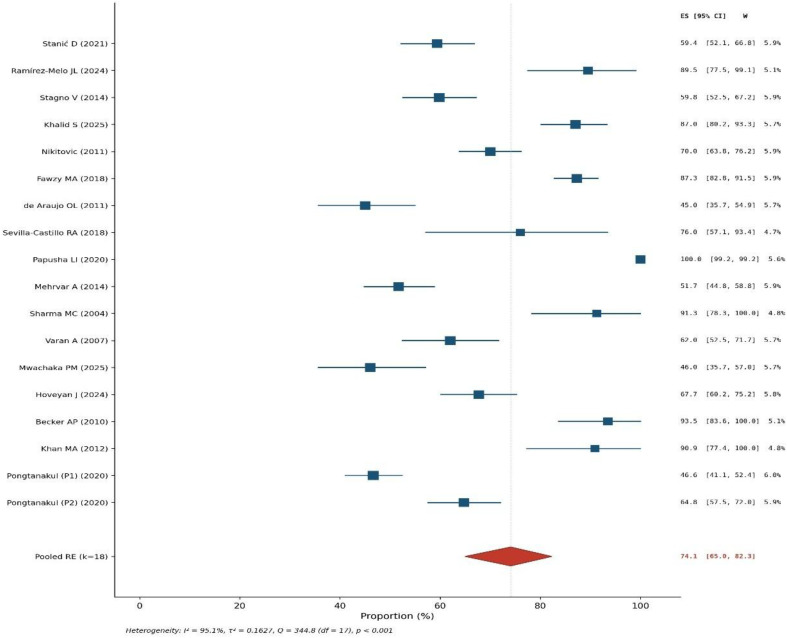
Forest plot of pooled 5-year overall survival (all studies).

Five-year OS data were extractable from 18 study populations. Random-effects meta-analysis yielded a pooled 5-year OS of 74.1% (95% CI: 65.0-82.3%). Heterogeneity was very high (I² = 95.1%, Q = 347.0, *p* < 0.001). Given the substantial heterogeneity in tumor biology, patient demographics, and study populations across the included glioma entities, the pooled survival estimates are best interpreted as descriptive indicators of overall trends in LMIC glioma care (**Figure 3**).

#### 3.7.2 LGG subgroup

**Figure 4:**
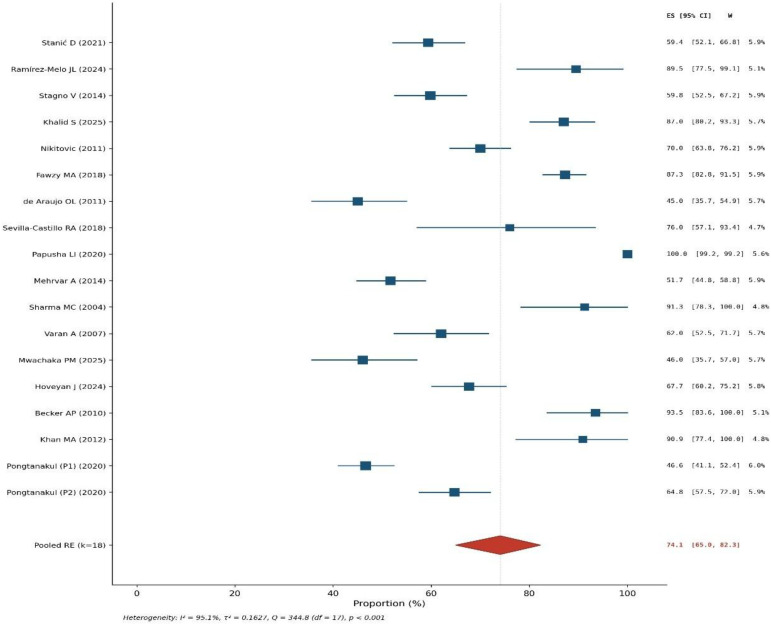
Forest plot of 5-year overall survival for low-grade gliomas.

Fifteen populations contributed LGG-specific data. Pooled 5-year OS was 87.8% (95% CI: 79.6-94.2%), I² = 89.1% (**Figure 4**). Mwachaka et al. (Kenya, 46.0%) was an outlier; excluding it increased the estimate to 90.2% (95% CI: 84.8-94.7%, I² = 76.6%).

#### 3.6.3 HGG subgroup

**Figure 5:**
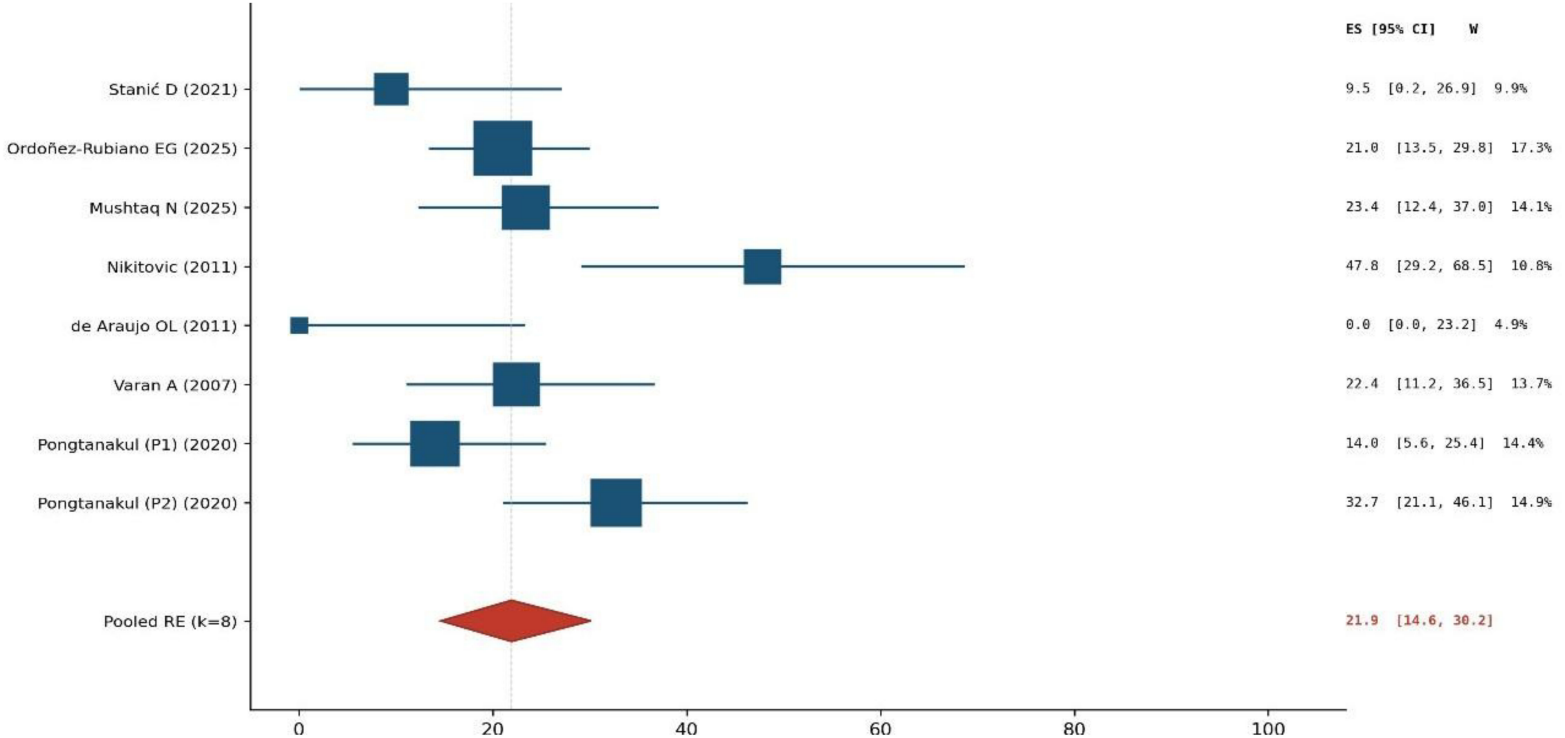
Forest plot of 5-year overall survival for high-grade gliomas.

Eight populations contributed HGG data. Pooled 5-year OS was 21.9% (95% CI: 14.6-30.2%), I² = 61.3% (**Figure 5**). GTR was a significant prognostic factor: Mushtaq et al. reported 2-year OS of 80% for GTR versus 8.1% for STR (*p* = 0.001).

#### 3.6.4 Event-free and progression-free survival

EFS/PFS data from 7 studies were heterogeneous, precluding pooling. Five-year EFS ranged from 10.0% (Khalid et al.) to 79.3% (Ramírez-Melo et al.). BRAF V600E was associated with lower 2-year PFS (30% vs 66%, *p* = 0.001).

### 3.7 Subgroup analyses

#### 3.7.1 Glioma-only studies

Restricting to 10 glioma-only studies increased pooled 5-year OS to 86.2% (95% CI: 73.6-95.4%, I² = 93.5%).

#### 3.7.2 Pediatric versus adult

Sixteen pediatric populations yielded pooled 5-year OS of 71.9% (95% CI: 62.2-80.6%, I² = 95.1%). Adult studies contributed insufficient 5-year OS data for formal pooling; individual cohorts reported mean OS of 14.1-39.8 months by histological subtype.

### 3.8 Meta-regression

Weighted least-squares meta-regression was performed to explore potential moderators of between-study heterogeneity in pooled 5-year overall survival.

#### 3.8.1 Publication year

**Figure 6:**
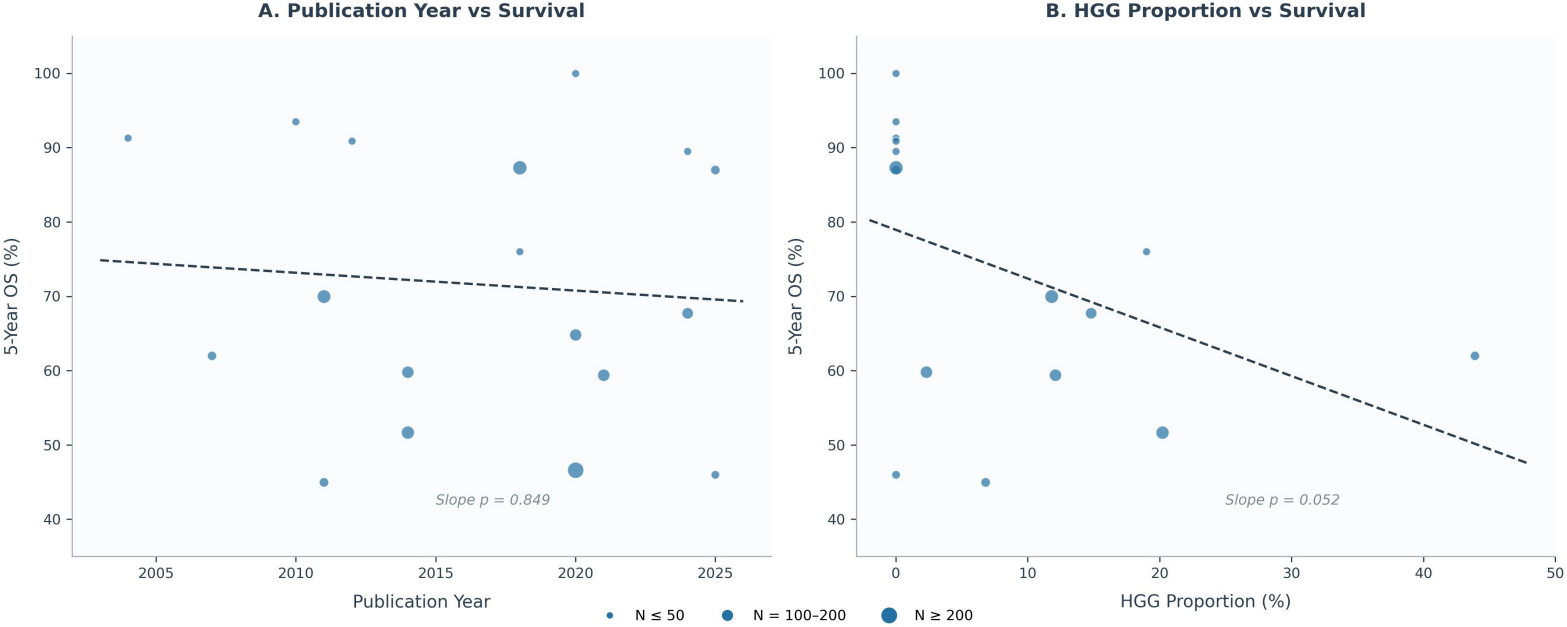
Meta-regression plots: **(A)** Publication year; **(B)** HGG proportion.

Publication year was not a significant predictor of 5-year OS (β = -0.0031, SE = 0.0161, z = -0.190, p = 0.849). No evidence of temporal improvement in survival across LMIC studies published between 2004 and 2025 (**Figure 6A**).

#### 3.8.2 HGG proportion

The proportion of HGG patients showed a borderline significant negative association with 5-year OS (β = -0.01753, p = 0.052). Each 10% increase in HGG proportion was associated with a decrease in Freeman-Tukey transformed survival, though this did not reach conventional significance (**Figure 6B**).

### 3.9 Publication bias

**Figure 7:**
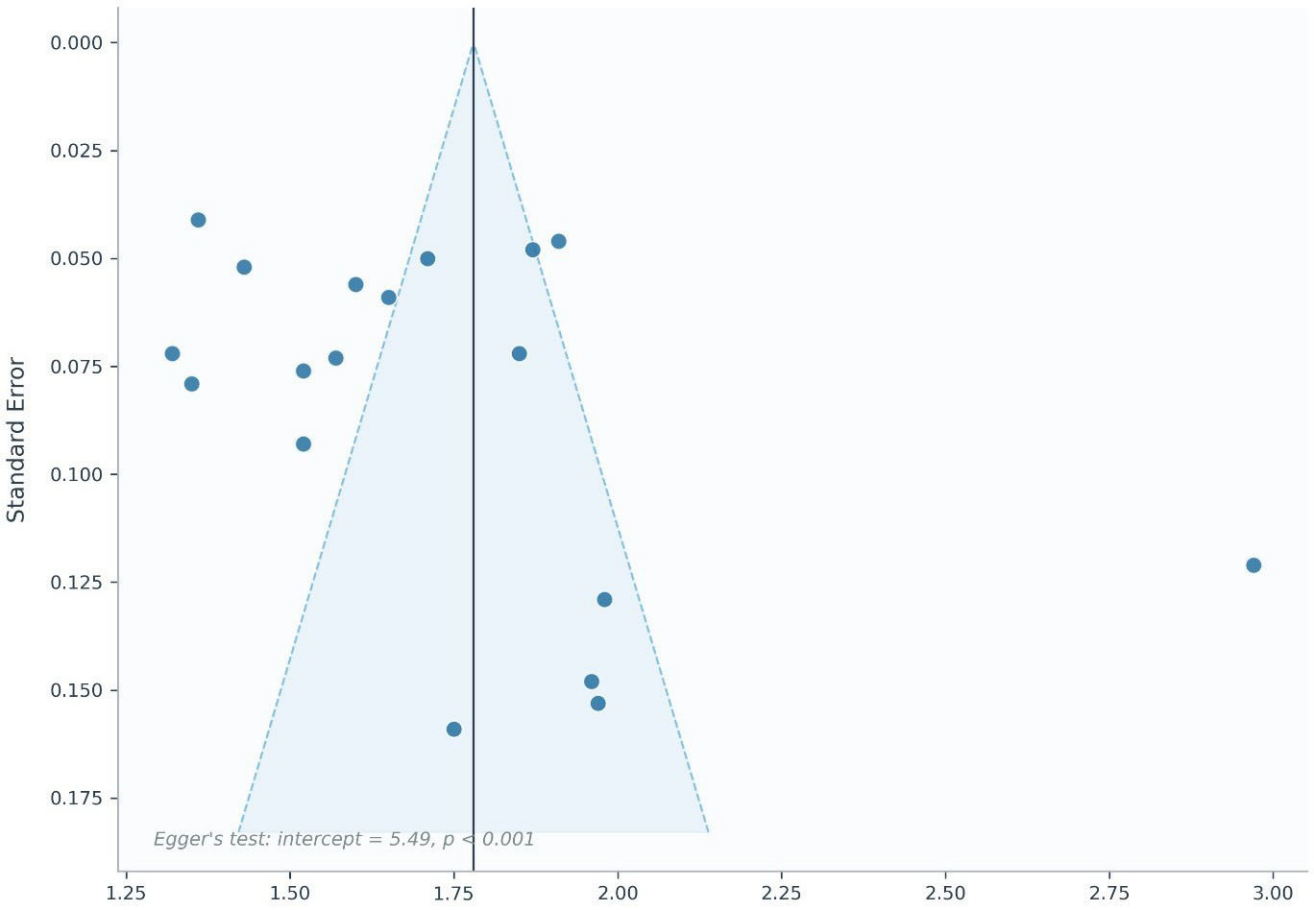
Funnel plot for assessment of publication bias.

Egger’s test detected significant funnel plot asymmetry (intercept = 5.49, *p* < 0.001), suggesting potential publication bias or small-study effects (**Figure 7**). However, this asymmetry likely reflects clinical heterogeneity rather than selective publication, given the high I² and diversity of included studies.

### 3.10 Sensitivity analysis

**Figure 8:**
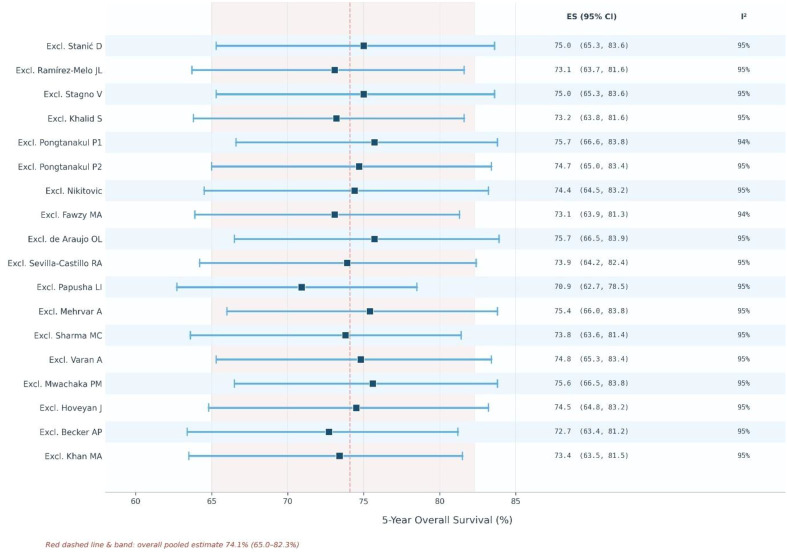
Leave-one-out sensitivity analysis.

Leave-one-out analysis demonstrated robustness of the pooled estimate (range: 70.9–75.7%) (**Figure 8**). Papusha et al. (OS = 100%) had the largest influence, lowering the estimate by 3.2 percentage points when excluded. I² remained above 93% in all iterations.

### 3.11 Quality assessment

The quality of the included studies ranged from low to high according to the Newcastle-Ottawa Scale (NOS). The majority of studies were of moderate quality (18/30, 60%), followed by high quality (10/30, 33.3%), while only two studies (6.7%) were rated as low quality: Baquero-Herrera et al. (25) and Khan et al. (10), each scoring 3 out of 9 stars. The highest-scoring studies were de Araujo et al. (27), Nikitovic et al. (32), and Stanić et al. (13), each achieving 8 out of 9 stars. The median NOS score across all studies was 6 (range: 3–8).

**Figure 9:**
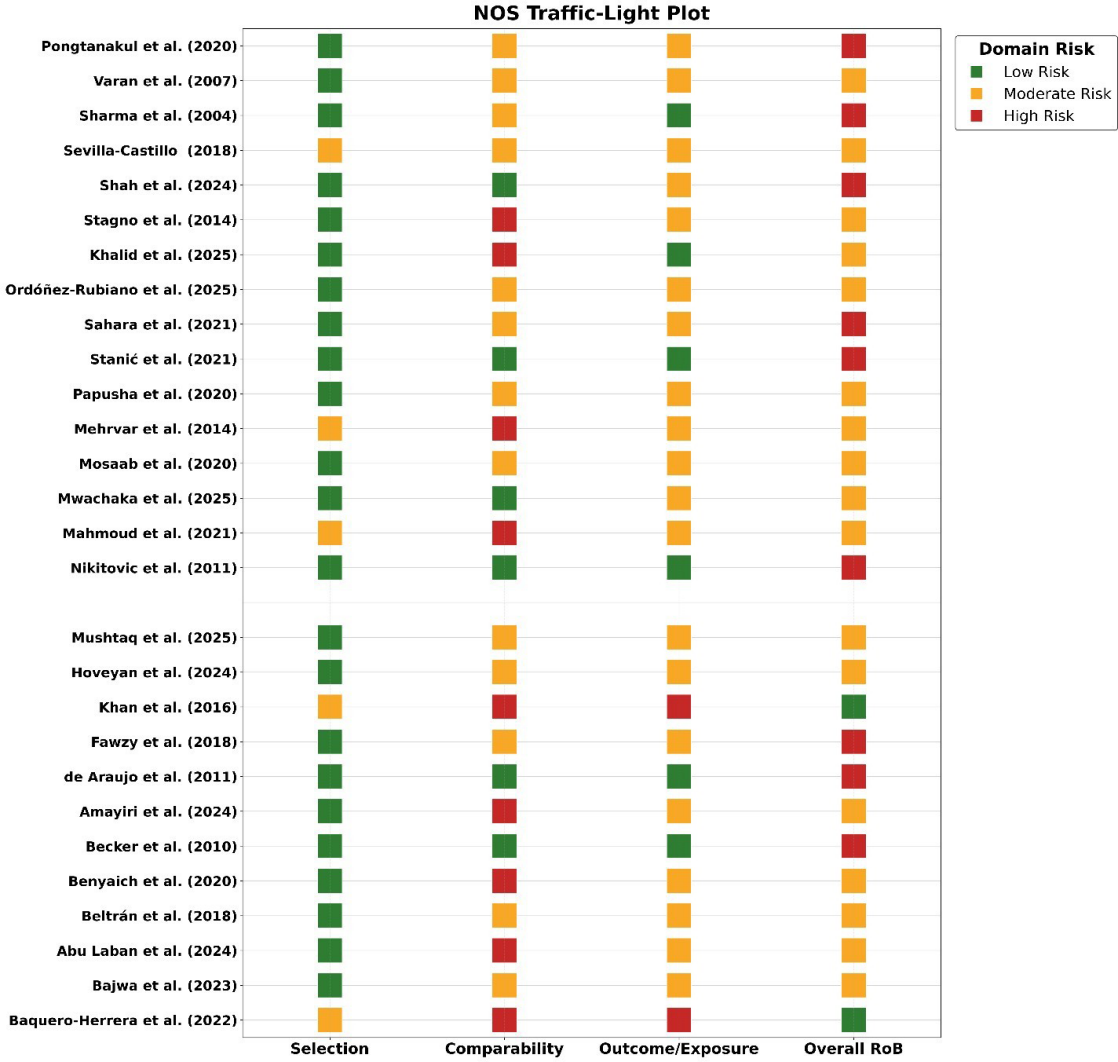
Newcastle-Ottawa Scale quality assessment summary by domain.

Regarding the individual domains, the Comparability domain was the weakest across the included studies: 10 studies (33.3%) scored 0 out of 2 stars, primarily due to the absence of a control or comparison group and the lack of multivariate adjustment for confounders. Only five studies (16.7%) achieved full comparability scores (2/2) (13, 22, 23, 27, 32). In the Selection domain, most studies scored 3 out of 4 stars (21/30, 70.0%), and three studies achieved full marks (4/4) (14, 29, 37). No study received a star for the selection of a non-exposed cohort, as most were single-arm case series or retrospective cohort designs without an external comparison group. In the Outcome domain, the majority of studies (28/30, 93.3%) scored at least 2 out of 3 stars, and six studies achieved full marks (3/3) (13, 19, 27, 28, 32, 35). The most common limitations in this domain were inadequate follow-up length and incomplete follow-up of cohorts. A summary of the NOS quality assessment domains is shown in (**Figure 9**), and the risk of bias (ROB) is displayed in **Supplementary Figure 1**. The detailed scoring for each study is presented in **Table 3**.

**Table 3:** Newcastle-ottawa scale (NOS) quality assessment summary.

Newcastle-ottawa scale (NOS) quality assessment summary					
Study	Selection (max 4★)	Comparability (max 2★)	Outcome (max 3★)	Total score (max 9★)	Quality rating
Baquero-Herrera et al. (25)	★★☆☆ (2/4)	☆☆ (0/2)	★☆☆ (1/3)	3/9	Low
Bajwa et al. (8)	★★★☆ (3/4)	★☆ (1/2)	★★☆ (2/3)	6/9	Moderate
Abu Laban et al. (12)	★★★☆ (3/4)	☆☆ (0/2)	★★☆ (2/3)	5/9	Moderate
Beltrán et al. (9)	★★★☆ (3/4)	★☆ (1/2)	★★☆ (2/3)	6/9	Moderate
Benyaich et al. (11)	★★★☆ (3/4)	☆☆ (0/2)	★★☆ (2/3)	5/9	Moderate
Becker et al. (28)	★★★☆ (3/4)	★☆ (1/2)	★★★ (3/3)	7/9	High
Amayiri et al. (18)	★★★☆ (3/4)	☆☆ (0/2)	★★☆ (2/3)	5/9	Moderate
de Araujo et al. (27)	★★★☆ (3/4)	★★ (2/2)	★★★ (3/3)	8/9	High
Fawzy et al. (29)	★★★★ (4/4)	★☆ (1/2)	★★☆ (2/3)	7/9	High
Khan et al. (10)	★★☆☆ (2/4)	☆☆ (0/2)	★☆☆ (1/3)	3/9	Low
Hoveyan et al. (26)	★★★☆ (3/4)	★☆ (1/2)	★★☆ (2/3)	6/9	Moderate
Mushtaq et al. (16)	★★★☆ (3/4)	★☆ (1/2)	★★☆ (2/3)	6/9	Moderate
Nikitovic et al. (32)	★★★☆ (3/4)	★★ (2/2)	★★★ (3/3)	8/9	High
Mahmoud et al. (21)	★★☆☆ (2/4)	☆☆ (0/2)	★★☆ (2/3)	4/9	Moderate
Mwachaka et al. (23)	★★★☆ (3/4)	★★ (2/2)	★★☆ (2/3)	7/9	High
Mosaab et al. (17)	★★★☆ (3/4)	★☆ (1/2)	★★☆ (2/3)	6/9	Moderate
Mehrvar et al. (31)	★★☆☆ (2/4)	☆☆ (0/2)	★★☆ (2/3)	4/9	Moderate
Papusha et al. (33)	★★★☆ (3/4)	★☆ (1/2)	★★☆ (2/3)	6/9	Moderate
Stanić et al. (13)	★★★☆ (3/4)	★★ (2/2)	★★★ (3/3)	8/9	High
Sahara et al. (14)	★★★★ (4/4)	★☆ (1/2)	★★☆ (2/3)	7/9	High
Ordóñez-Rubiano et al. (15)	★★★☆ (3/4)	★☆ (1/2)	★★☆ (2/3)	6/9	Moderate
Khalid et al. (19)	★★★☆ (3/4)	☆☆ (0/2)	★★★ (3/3)	6/9	Moderate
Stagno et al. (20)	★★★☆ (3/4)	☆☆ (0/2)	★★☆ (2/3)	5/9	Moderate
Shah et al. (22)	★★★☆ (3/4)	★★ (2/2)	★★☆ (2/3)	7/9	High
Khan et al. (30)	★★☆☆ (2/4)	☆☆ (0/2)	★★☆ (2/3)	4/9	Moderate
Sevilla-Castillo et al. (34)	★★☆☆ (2/4)	★☆ (1/2)	★★☆ (2/3)	5/9	Moderate
Sharma et al. (35)	★★★☆ (3/4)	★☆ (1/2)	★★★ (3/3)	7/9	High
Varan et al. (36)	★★★☆ (3/4)	★☆ (1/2)	★★☆ (2/3)	6/9	Moderate
Ramírez-Melo et al. (60)	★★★☆ (3/4)	★☆ (1/2)	★★☆ (2/3)	6/9	Moderate
Pongtanakul et al. (37)	★★★★ (4/4)	★☆ (1/2)	★★☆ (2/3)	7/9	High

Quality Rating: Low (0-3★) | Moderate (4-6★) | High (7-9★).

## 4 Discussion

### 4.1 Summary of evidence

The global incidence of malignant brain tumors is estimated at 4.25 cases per 100,000 person-years, with notable regional variation. Rates are higher in Europe (6.76 per 100,000) compared to Africa (2.81 per 100,000), and similarly greater in HICs (6.29 per 100,000) than in LMICs (4.81 per 100,000) (38). The lower reported incidence in LMICs likely reflects limitations in diagnostic capacity and case detection, rather than a true reduction in disease burden (39). A more recent analysis estimated 322,000 new cases of brain and CNS tumors globally in 2022, with the highest incidence rates in Northern America (5.46 per 100,000), Western Europe (5.56 per 100,000), and Eastern Asia (3.95 per 100,000), while Africa had relatively lower rates (40). These disparities in reported incidence highlight the fundamental challenge of underdiagnosis and underreporting in LMICs, which subsequently affects resource allocation and health policy planning for glioma care.

The male predominance observed in our review (58.6% males; male-to-female ratio of 1.42:1) is consistent with the well-documented sex differences in glioma epidemiology. Population-based studies have consistently demonstrated that most glioma histologies occur with a 50% higher incidence in males (41). Data from the Central Brain Tumor Registry of the United States showed that among 294,886 patients with primary malignant gliomas, 55.9% were males and 44.1% were females (42).

A notable finding of this review is the relatively young pooled mean age of approximately 45.1 years for HGGs, which differs considerably from the median age at diagnosis reported in HICs, where glioblastoma typically presents in patients older than 60 years (42). This young age distribution likely reflects the higher proportion of the younger population demographics of LMICs, and the relatively low life expectancy in some settings, which may result in underrepresentation of older adults with glioma.

Surgical intervention was the most consistently documented treatment modality, with 94.9% of patients undergoing some form of surgery. This high rate of surgical management underscores the central role of surgery in glioma treatment across LMIC settings. The extent of resection is a critical prognostic factor: a meta-analysis of 37 studies demonstrated that GTR was significantly associated with a lower relative risk for mortality at 1 and 2 years compared with subtotal resection, with a dose-dependent reduction in mortality observed with increasing extent of resection (43). The mean overall survival for GTR versus incomplete resection has been reported at 28.7 versus 13.5 months (44). A landmark study of 416 glioblastoma patients found that a significant survival advantage was associated with resection of 98% or more of the tumor volume (median survival 13 months) compared with less than 98% resection (median survival 8.8 months) (45). However, achieving GTR in LMICs remains challenging, largely due to limitations in neurosurgical infrastructure and the restricted availability of advanced intraoperative technologies that support surgical precision (46).

Access to adjuvant therapies was notably restricted in our review, with only 53.6% of patients receiving radiotherapy and 41.7% receiving chemotherapy. This is a significant concern given the established survival benefits of combined chemoradiation. The landmark phase III trial by Stupp et al. demonstrated that adding temozolomide to radiotherapy for newly diagnosed glioblastoma resulted in a median survival of 14.6 months compared with 12.1 months with radiotherapy alone, with a two-year survival rate of 26.5% versus 10.4% (47).

The Stupp protocol has become the gold standard for glioblastoma management, with real-world evidence demonstrating median overall survival of 16.0 months and 2-year overall survival of 30.7% in treated cohorts. The finding that nearly half of patients in our review did not receive radiotherapy and two-thirds did not receive chemotherapy suggests that a substantial proportion of glioma patients in LMICs are managed suboptimally relative to established standards of care, which directly contributes to inferior survival outcomes.

The limited access to adjuvant therapies in LMICs has been attributed to several factors. Glioblastoma treatment is expensive, and unregulated radiation and chemotherapy costs can expose patients to financial hardships and lead to treatment discontinuation (39). Radiation therapy facilities are unavailable in many poor-resource settings (39). A study from a low-income country demonstrated that the median survival for a radiotherapy-alone group was 5.3 months compared with 14.8 months for combined radiotherapy/temozolomide, suggesting that when temozolomide is available, outcomes comparable to those from developed nations can be expected if healthcare delivery is carefully planned (48).

One of the most critical gaps identified in this review is the limited integration of molecular diagnostics, with only 26.7% of included studies reporting molecular markers such as IDH mutation status or 1p/19q co-deletion. The 2021 WHO Classification of Central Nervous System Tumors has placed molecular markers at the center of glioma diagnosis and classification (49).

Markers such as MGMT, EGFR, IDH, 1p/19q, ATRX, TERT, FGFR-TACC, and BRAF are now used to classify brain tumors and influence management decisions, with several also serving as therapeutic targets (50). IDH-mutated tumors exhibit better prognosis throughout every grade of glioma, and IDH mutation status is considered an essential diagnostic criterion (50). The IDH1/2 inhibitor vorasidenib has been shown in a phase 3 clinical trial to prolong progression-free survival (27.7 months vs. 11.1 months) in patients with IDH-mutant grade II gliomas (51). Without access to these molecular diagnostics, patients in LMICs cannot benefit from the precision medicine approaches that are increasingly standard in HICs, resulting in suboptimal risk stratification, treatment selection, and prognostication.

The survival outcomes in our review reflected substantial heterogeneity across studies, while stratification by tumor grade demonstrated marked prognostic differences, with low-grade gliomas (LGGs) showing substantially better survival outcomes compared with high-grade gliomas (HGGs). The pooled 5-year overall survival reached 87.8% for LGGs, whereas HGGs demonstrated a markedly lower pooled 5-year overall survival of only 21.9%. These findings underscore the strong influence of tumor biology and grade on long-term outcomes and may partially explain the broad variability in survival estimates observed across included studies. Evidence has shown that the relationship between mortality and incidence in glioma patients is driven by socioeconomic and sociodemographic factors, with studies clearly demonstrating that survival is significantly higher among patients with high socioeconomic status (52). Socioeconomic factors influence glioma outcomes through multiple mechanisms, including general access to health systems, access to specialized diagnostic and therapeutic resources, and potential exposure to environmental or occupational hazards (53).

Beyond the clinical findings summarized above, the results of this review must be interpreted within the broader context of LMIC-specific structural barriers that fundamentally shape glioma care delivery. A critical challenge is the severe shortage of neurosurgical workforce in LMICs. Globally, more than 5 million patients in LMICs who would benefit from a neurosurgery operation do not receive this care annually, and the neurosurgeon-to-population ratio remains alarmingly low, with many countries in Africa and Southeast Asia falling below the minimum target of 0.5 neurosurgeons per 100,000 populations (54, 55). In Africa, the workforce deficit is particularly acute, with an estimated density of only 0.15 neurosurgeons per million people, and only 32 of 54 countries offer neurosurgical training programs (56). Approximately 85.6% of practicing pediatric neurosurgeons globally operate in high-income countries, and those in LMICs are significantly less likely to have obtained formal graduate-level subspecialty training in pediatric neurosurgery (57).

Radiotherapy infrastructure shortages remain a major contributor to the treatment disparities observed across LMICs. Among the 139 LMICs globally, 55 lack any radiotherapy facilities, while the other 84 countries continue to face substantial shortages, operating with only a fraction of the radiotherapy capacity required to meet clinical demand (58). Furthermore, the lack of universal health coverage in many LMICs means that the cost of essential chemotherapeutic agents such as temozolomide, while available as generic formulations, remains prohibitively expensive relative to household income levels.

The absence of standardized treatment protocols, cancer registries, and multidisciplinary tumor boards in many LMIC settings results in fragmented care pathways where patients may receive surgery without subsequent adjuvant therapy, or where molecular diagnostics are unavailable to guide treatment selection. The limited integration of molecular diagnostics in LMICs, with only 26.7% of studies in our review reporting molecular markers, is particularly concerning given the 2021 WHO classification’s emphasis on molecular characterization. The primary barriers to molecular testing in LMICs include the high capital costs of next-generation sequencing and fluorescence *in situ* hybridization platforms, insufficient laboratory infrastructure, shortage of trained molecular pathologists, and lack of quality assurance programs (59).

This systematic review synthesized data from 30 studies across 17 low- and middle-income countries (LMICs), encompassing 3,463 glioma patients, and represents one of the most comprehensive analyses of glioma management in resource-limited settings to date. The findings reveal a landscape characterized by male predominance, heavy reliance on surgical intervention, limited access to adjuvant therapies, restricted molecular diagnostics, and heterogeneous survival outcomes. These observations stand in contrast to the well-established diagnostic and treatment paradigms in HICs and underscore the persistent global disparities in neuro-oncological care.

### 4.2 Strength points

This systematic review has several notable strengths. First, it provides a comprehensive and multi-regional overview of glioma management in LMICs, incorporating data from 30 studies across 17 countries, which enhances the generalizability of the findings. Second, the review was conducted in accordance with PRISMA guidelines and was registered in the PROSPERO database, ensuring methodological rigor and transparency. Third, the inclusion of both pediatric and adult populations provides a broader perspective on glioma management across the lifespan in resource-limited settings. Fourth, quality assessment was performed using the Newcastle-Ottawa Scale, with two independent reviewers conducting blinded assessments, adding objectivity to the evaluation. Fifth, the review systematically examined multiple dimensions of glioma care, including diagnosis, surgical management, adjuvant therapies, molecular diagnostics, and survival outcomes, providing a holistic view of the challenges facing LMICs.

### 4.3 Limitations

This review has several limitations that should be acknowledged. First, substantial methodological and clinical heterogeneity existed across the included studies, with marked variation in tumor biology, patient populations, study designs, treatment protocols, and outcome reporting, which limits the comparability of pooled estimates. Second, the search strategy was restricted to PubMed and Scopus. Third, reporting was inconsistent for several key variables, including extent of resection, treatment protocols, molecular markers, and follow-up duration, which limited precise quantification and comparison. Fourth, the pooled mean age and survival estimates should be interpreted cautiously, as they were derived from studies with heterogeneous reporting formats and patient populations. Fifth, regional subgroup analyses were limited by the relatively small number of studies and inconsistent reporting across LMIC regions. Finally, the limited reporting of molecular data in most studies precluded a meaningful subgroup analysis by molecular markers, which is increasingly relevant in the era of molecular-based glioma classification.

## 5 Conclusion

This systematic review, synthesizing data from 30 studies across 17 LMICs encompassing 3,463 glioma patients, reveals significant disparities in glioma management and outcomes compared with high-income countries. While surgical intervention remains nearly universal (94.9%), access to adjuvant therapies is markedly restricted, with only 53.6% of patients receiving radiotherapy and 41.7% receiving chemotherapy, far below the standards established by landmark trials demonstrating the survival benefits of combined chemoradiation. Addressing these gaps requires a multifaceted approach, including investment in radiotherapy infrastructure, ensuring affordable access to essential chemotherapeutic agents such as temozolomide, expanding molecular diagnostic capabilities, and establishing standardized treatment protocols adapted to resource-limited settings. Future multicenter prospective studies from LMICs are needed to generate higher-quality evidence that can inform context-specific clinical guidelines and health policy. Ultimately, closing the gap in glioma care between LMICs and high-income countries is essential to achieving global equity in neuro-oncological outcomes.

## Data Availability

The original contributions presented in the study are included in the article/**Supplementary Material**. Further inquiries can be directed to the corresponding author.
