# Supplementary figures and images for: Glioma management and outcome in low-middle-income countries: a systematic review
Source: Front Oncol. 2026 Jun 12;16:1856016. doi: 10.3389/fonc.2026.1856016 (PMC13303666; doi:10.3389/fonc.2026.1856016)

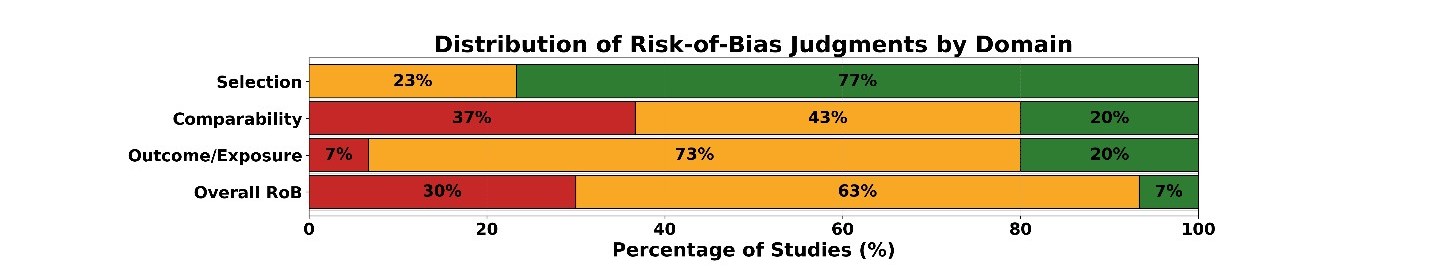

Supplement: Supplementary file 1 [file Image1.jpeg]
